# A comparison between antibiotic utilisation in public and private community healthcare in Malaysia

**DOI:** 10.1186/s12889-023-17579-3

**Published:** 2024-01-03

**Authors:** Audrey Huili Lim, Norazida Ab Rahman, Siti Nur Su’aidah Nasarudin, Tineshwaran Velvanathan, Mary Chok Chiew Fong, Abdul Haniff Mohamad Yahaya, Sheamini Sivasampu

**Affiliations:** 1 Institute for Clinical Research, National Institutes of Health, Shah Alam, Malaysia; 2 Pharmacy Research & Development Branch, Pharmacy Policy & Strategic Planning Division, Pharmaceutical Services Programme, Ministry of Health, Petaling Jaya, Malaysia

**Keywords:** Antibiotic utilisation, Primary care, Community health, Defined daily dose, DU90, AWaRe

## Abstract

**Background:**

There are two parallel systems in Malaysian primary healthcare services: government funded public primary care and privately-owned practices. While there have been several studies evaluating antibiotic utilisation in Malaysian public healthcare, there is a lack of literature on the use of antibiotics in the private sector. There is a dire need to evaluate the more recent performance of public vs. private community healthcare in Malaysia. As such, this study aimed at measuring and comparing the utilisation of antibiotics in the public and private community healthcare sectors of Malaysia in 2018–2021.

**Methods:**

This study was a retrospective analysis of antibiotic utilisation in Malaysian primary care for the period of 1 January 2018 until 31 December 2021 using the nationwide pharmaceutical procurement and sales data from public and private health sectors. Rates of antibiotic utilisation were reported as Defined Daily Doses per 1000 inhabitants per day (DID) and stratified by antibiotic classes. The secondary analysis included proportions of AWaRe antibiotic category use for each sector and proportion of antibiotic utilisation for both sectors.

**Results:**

The overall national antibiotic utilisation for 2018 was 6.14 DID, increasing slightly to 6.56 DID in 2019, before decreasing to 4.54 DID in 2020 and 4.17 DID in 2021. Private primary care antibiotic utilisation was almost ten times higher than in public primary care in 2021. The public sector had fewer (four) antibiotic molecules constituting 90% of the total antibiotic utilisation as compared to the private sector (eight). Use of Access antibiotics in the public sector was consistently above 90%, while use of Access category antibiotics by the private sector ranged from 64.2 to 68.3%. Although use of Watch antibiotics in the private sector decreased over the years, the use of Reserve and ‘Not Recommended’ antibiotics increased slightly over the years.

**Conclusion:**

Antibiotic consumption in the private community healthcare sector in Malaysia is much higher than in the public sector. These findings highlight the need for more rigorous interventions targeting both private prescribers and the public with improvement strategies focusing on reducing inappropriate and unnecessary prescribing.

**Supplementary Information:**

The online version contains supplementary material available at 10.1186/s12889-023-17579-3.

## Introduction

Antibiotics are the most commonly prescribed drugs worldwide [1, 2]. The extensive utilization of antibiotics is believed to have prolonged the average lifespan by two decades, causing a shift in the focus of diseases from communicable to non-communicable ones. The global public health challenge of antimicrobial resistance has been exacerbated by the excessive usage of antibiotics worldwide. Overprescription of antibiotics is linked to a higher risk of adverse effects, more frequent revisits to healthcare facilities, and the unnecessary medicalization of self-limiting conditions. The overprescription of antibiotics is particularly prevalent in primary care, where viral infections are the primary cause of most illnesses [3, 4].

The World Health Organization (WHO) Expert Committee on Selection and Used of Essential Medicines developed the AWaRe Classification of antibiotics in 2017 as a tool to support antibiotic stewardship efforts at local, national, and global levels. The AWaRe classification categorizes antibiotics into three groups: Access, Watch, and Reserve, and emphasizes the importance of appropriate antibiotics use by taking into consideration the impact of different antibiotics and antibiotic classes on antimicrobial resistance. The Access group consists of first and second choices of empirical treatment of the most common infections. The Watch group antibiotics are indicated for a few, specific infectious conditions as they are more susceptible to antibiotic resistance and/or have higher toxicity concern. The Reserve group antibiotics are the “last resort” for highly selected patients (e.g. those with serious or life-threatening infections due to multidrug resistant bacteria) and require close monitoring [5, 6].

In order to promote responsible use of antibiotics, evidence shows that at least 60% of national antibiotic consumption should consists of Access antibiotics for not only judicious use of antibiotics, but also reduced costs and increased access. One of the health-related targets of the sustainable development goals includes a target to achieve this threshold by 2023 [7].

Malaysia has a dual healthcare system with parallel public and private primary care health sectors [7]. The primary care level functions as the first point of contact care in a community setting [8]. A public clinic is usually staffed with doctors, nurses, pharmacists, and some other professions such as dietitians or physiotherapists, while the workforce of private clinics mainly consists of doctors and non-certified nursing aides where approximately 75% of these clinics are solo practices [9]. Federal revenue and taxation fund healthcare in the public sector while the private sector is funded mainly through out-of-pocket payments from patients, private health insurance and employee health benefits schemes. Private primary healthcare is mainly concentrated in the urban, affluent areas, focusing on acute conditions and curative care. Malaysia has yet to implement dispensing separation in the private sector; therefore, antibiotics can be prescribed and dispensed directly from doctors in the private clinics [10, 11]. Antibiotics can only be purchased from retail pharmacies with a valid prescription. Although treatment guidelines for the management of infectious diseases or antibiotic treatment in primary care settings is available to provide recommendations for reasonable antibiotic usage, guideline adoption varies across practices in Malaysia [12]. In addition, the Ministry of Health (MOH) Malaysia has initiated a local antimicrobial stewardship (AMS) program for implementation at hospitals and primary care to encourage stewardship activities in all healthcare facilities in Malaysia [13].

There is a need to evaluate the more recent data on overall antibiotic use in primary care facilities in Malaysia to understand the utilisation pattern and for enhancement of AMS initiative. Furthermore, there remains a lack of data particularly with respect to the use of AWaRe classification to assess the antibiotics utilisation in public and private primary care facilities. As such, this study aimed at measuring patterns of antibiotic utilisation in public and private sector at primary care level for the period 2018–2021.

## Methods

This study was reported in accordance to the STrengthening the Reporting of OBservational studies in Epidemiology (STROBE) checklist (Supplementary Table S1).

### Study design and setting

This study was a retrospective analysis of aggregated sales data of antibiotics for primary care facilities in Malaysia for the period 2018–2021. This study used the national drug procurement data obtained from the database of the Pharmaceutical Services Programme, Ministry of Health Malaysia. The database includes medicine purchasing records of all government health facilities throughout Malaysia. Detailed information about the database is described elsewhere [14]. Data on private sector procurement was derived from sales data which represents approximately two-third of the total pharmaceutical market coverage in Malaysia. As antibiotics prescribed in private clinics can also be purchased in retail pharmacies, sales data for retail pharmacies (private sector) were also collected. Detailed description of the data source is described elsewhere [15]. The public primary care is comprised of over 1000 public health clinics nationwide whereas the private sector includes nearly 8000 private clinics (general practitioners) that provide outpatient care and approximately 3000 retail community pharmacies [16, 17]. Data on population size was obtained from the Department of Statistics Malaysia, based on the yearly population estimates of Malaysia [18].

### Antibiotics

Data on antibiotics was retrieved according to the 2020 WHO Anatomical Therapeutic Chemical (ATC) Classification System [17]. All antibiotics for systemic use (code: J01) were identified and grouped by antibiotic classes. A total of 14 antibiotic classes (aminoglycosides, amphenicols, carbapenems, cephaloporins, fluoroquinolones, glycopeptides, macrolides, nitrofurans, nitroimidazoles, penicillins, steroids, sulfonamides, tetracyclines, and others) and 76 antibiotic molecules were included in this study.

Antibiotics were also classified into the AWaRe categories: Access, Watch, Reserve, and Not Recommended.

### Statistical analysis

Data were converted into defined daily doses (DDDs) using the 2020 WHO ATC/DDD index. Antibiotic utilisation rates were calculated from the total DDD divided by the population and reported as DDD per 1000 inhabitants per day (DID) (Eq. 1) [18]. Percentage of antibiotics by antibiotic classes and AWaRe categories were calculated as utilisation per categories divided by total utilisation. Sensitivity analysis was performed by analysing data from private clinics only for antibiotic utilisation in the private sector. R version 4.1.0 was used to conduct data cleaning and analysis.

1 $$ \begin{aligned}&DDD\;per\;1000 \;inhabitants \;per\;day\\&= \frac{total\;dose\;of\;drug,\;by\;ATC \;code}{DDD \times population \times number \;of \;days } \times 1000 \end{aligned}$$

## Results

**Fig. 1:**
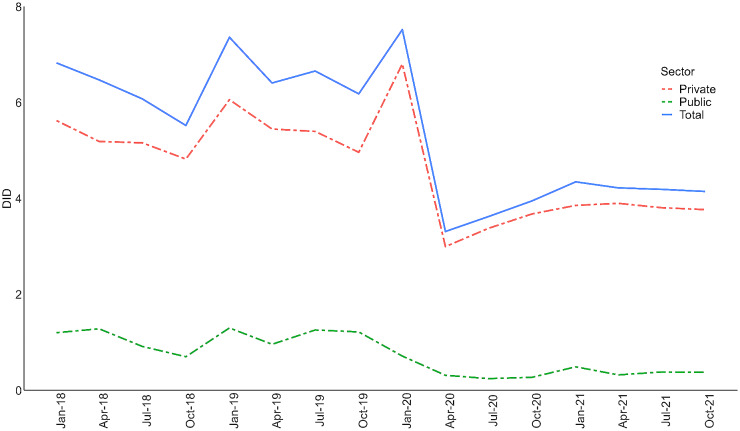
Overall trend of antibiotic utilisation in public and private sector during 2018-2021

In 2021, total antibiotics utilisation for primary care in Malaysia was 4.17 DIDs. The antibiotics utilisation rate in private sector was higher at 3.78 DIDs compared to public sector (0.39 DIDs). Between 2018 and 2021, both sectors had declines in the antibiotic utilisation rate. The utilisation rate in the public sector decreased by 61.4% (from 1.01 to 0.39 DIDs) while in private sector it decreased by 26.3% (from 5.13 to 3.78 DIDs). The trend in antibiotic utilisation rates between 2018 and 2021 are shown in Fig. 1 by quarterly periods. The utilisation of antibiotics fell substantially in May-August 2020 as compared with the earlier period of January 2018-April 2020. The rate started to increase again in 2021. The private sector accounted for at least 80% of the total antibiotic utilisation in primary care, with the ratio remaining stable over the four years.

**Fig. 2:**
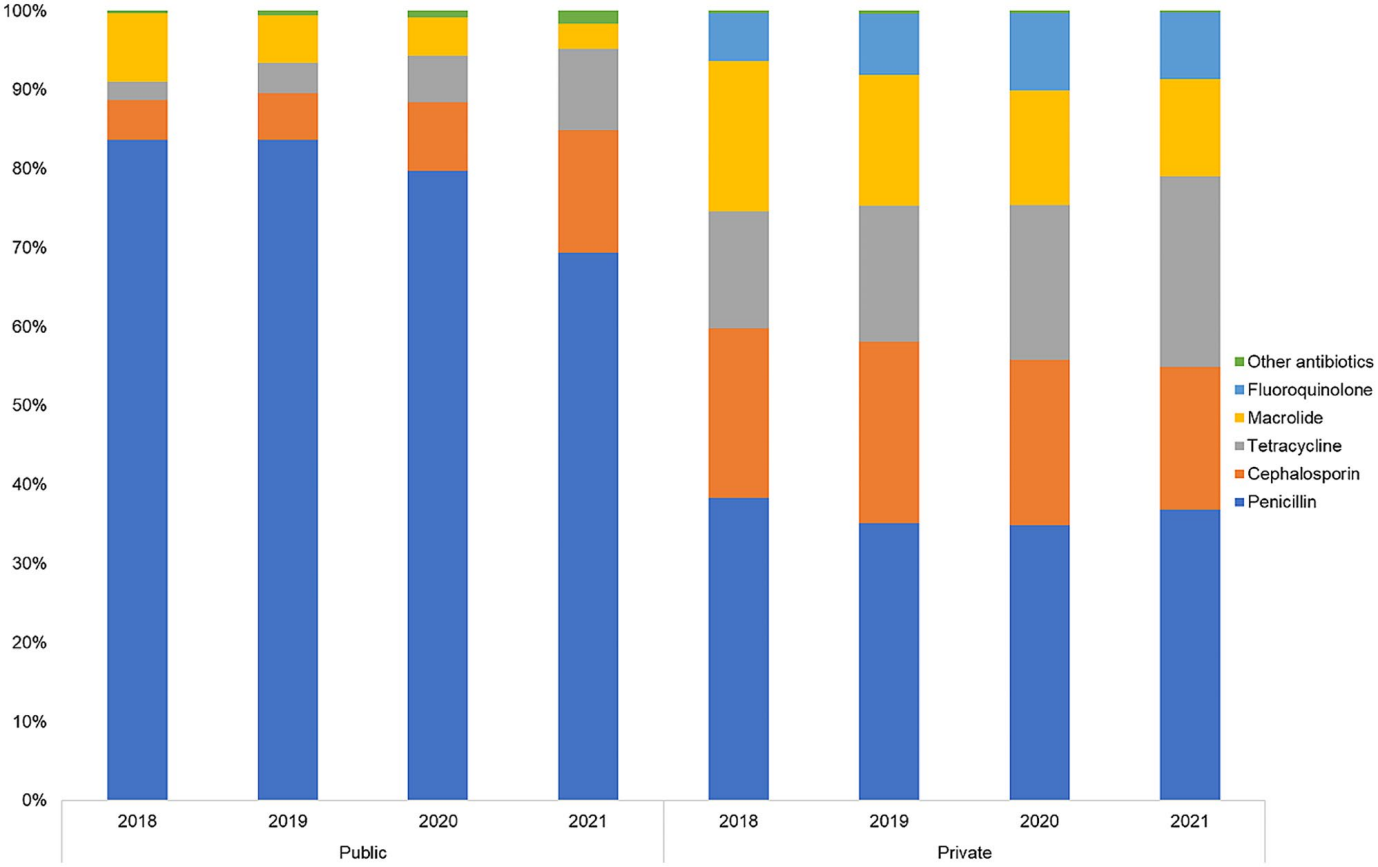
Antibiotic classes used in public and private sector by share of DDDs during 2018-2021

**Fig. 3:**
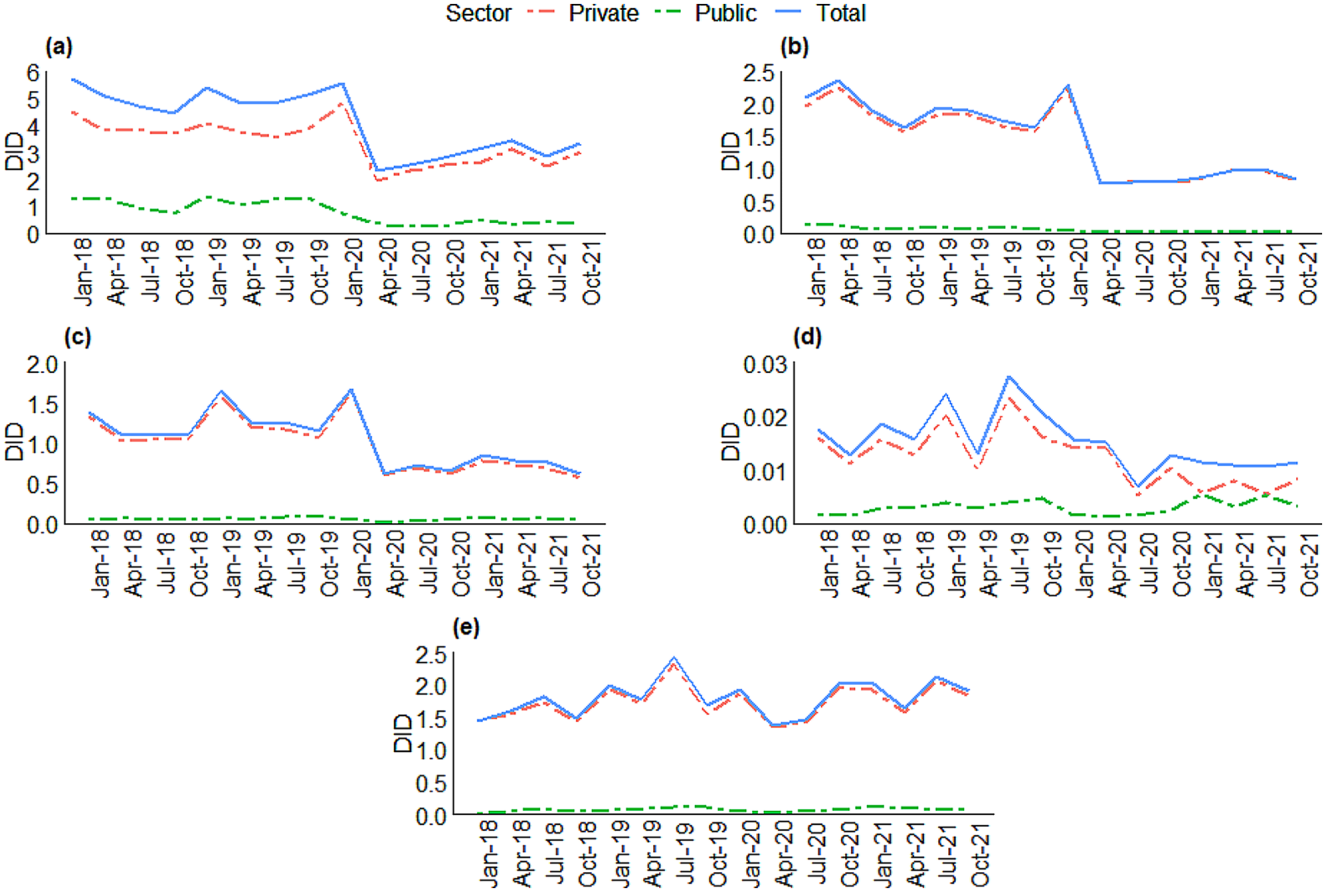
Trends of antibiotic utilisation in public and private sector during 2018–2021 for (**a**) penicillins; (**b**) macrolides; (**c**) cephalosporins; (**d**) sulfonamides; (**e**) tetracyclines

Distribution of antibiotic classes used in public and private sector are described in the percentage of all antibiotics (Fig. 2). Penicillins had the highest usage, accounting for 81% of antibiotics in public and 37% in private. Cephalosporins, macrolides, and tetracyclines took up a fair share of usage in the private sector, ranging between 15 and 41% of all antibiotics. The proportions of the use of these three classes in the public sector was much lesser than that in private. Figure 3 describes the trend in utilisation rates in DIDs for the five antibiotic classes used in both sectors: penicillins, macrolides, cephalosporins, sulfonamides, and tetracyclines. Utilisation of most antibiotics showed a decreasing trend between 2018 and 2021 for both public and private sectors, except for tetracyclines (75% increase in public and 20% increase in private) and cephalosporins (16% increase in public) (Fig. 3, Supplementary Table S2). Four antibiotic molecules constituted 90% of the total antibiotic utilisation (Drug Utilisation 90, DU90%) in the public sector while 8 antibiotic molecules constituted DU90% of antibiotics in the private sector (Supplementary Table S3).

**Fig. 4:**
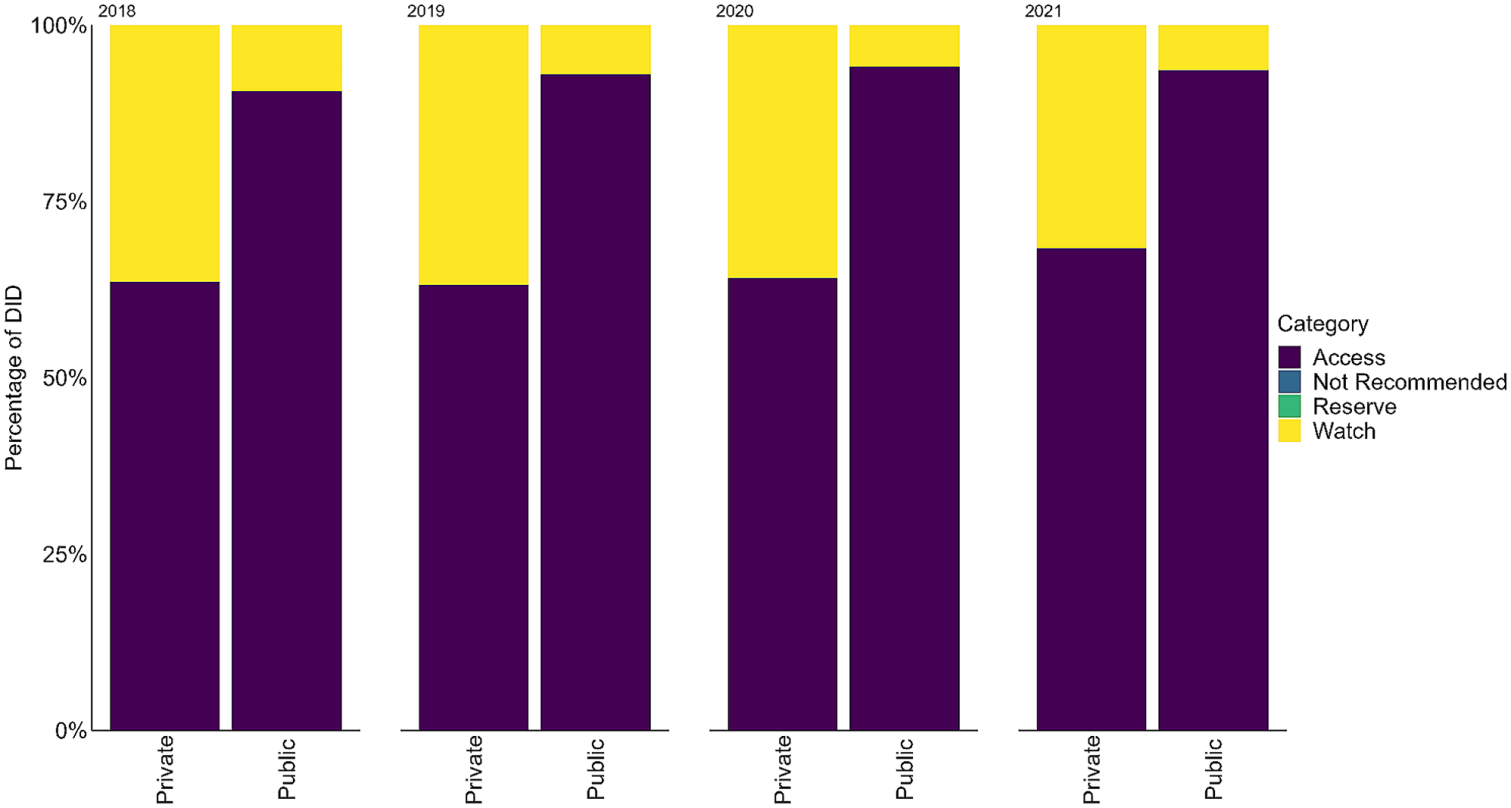
Proportions of different AWaRe categories of antibiotics for public and private sectors during 2018-2021

In the public sector, antibiotics from the Access group consistently accounted for at least 90% of the total antibiotics used (Fig. 4). The remaining proportion of antibiotics in the public sector was from the Watch category. For the private sector, the Access group antibiotics contributed 63–68% of the total antibiotics used during the period from 2018 to 2021. The Watch group antibiotics contributed one-third and there was minute use of antibiotics from the Reserve group (< 0.01%).

We compared antibiotic utilisation by type of primary care facilities. All primary care facilities in the public sector are clinics while private sector comprised of clinics and community pharmacies. Within the private sector, clinics accounted for approximately 80% of total antibiotic utilisation whereas pharmacies constituted 20% (Supplementary Table S4).

Sensitivity analysis showed that proportion of Access category antibiotics used in the private sector decreased slightly when only sales data from private clinics were included (62–65% compared to 63–68%). There were no changes in the antibiotic molecules that constituted DU90. However, there was a small difference in the distribution of classes, with macrolides forming a greater proportion of use compared to tetracyclines (Supplementary Figure S1).

## Discussion

This study provides the most recent and comprehensive estimates on systemic antibiotic use at the primary care level in Malaysia for the public and private sectors. While earlier studies may have described antibiotic utilisation in various small-scale settings within the Asian region, to our knowledge, none have used the WHO AWaRe classification to assess and compare antibiotic utilisation between public and private facilities. In this study, we quantified changes in antibiotic utilisation rates over a four-year period from 2018 to 2021 and compared antibiotic use patterns using the DDD metrics and AWaRe classification.

We observed a reduction in overall antibiotic use across the primary care facilities in Malaysia during the study period. The decreasing trend of antibiotic utilisation rates in both sectors is encouraging, but the decline was likely driven by the COVID-19 pandemic. A substantial reduction was seen particularly in 2020 which coincides with the time of the national lockdown and restrictions implemented in Malaysia due to COVID-19 which began in March 2020 [20]. Movement restrictions and COVID-19 concerns led to fewer patients seeking care at primary care facilities and reduced frequency of infectious disease or influenza-like illnesses, which may have affected the use of antibiotics during this period. The reduction in antibiotic utilisation during the pandemic period was seen in both public and private sectors, reflecting reduced overall stream of patients. Yang et al. also reported a decrease in antibiotic utilisation from an analysis of China’s national procurement data due to the COVID-19 pandemic [19]. In Japan, there was a 20% decrease in antimicrobial consumption in 2020 compared to the preceding year [20]. Similar findings were observed in Scotland and Canada by Malcolm et al. and Knight et al. [21, 22], while Nandi et al. reported that the global sales of four antibiotic group decreased in April and May 2020 compared to the pre-pandemic period [6].

The global goal of WHO is to have at least 60% share of antibiotics from the Access group [23]. Our findings showed that in primary care, both public and private sectors achieved this target. For the public sector in Malaysia, utilisation of antibiotics is restricted by the MOH formulary that determines the type of antibiotic formulations that can be prescribed in the public health facilities [24]. The formulary not only serves as a reference for medicines used in public MOH facilities but also functions as a policy and administrative approach to regulate and encourage rational and quality use of medicines in all MOH institutions. Drugs are evaluated for functionality and cost effectiveness before being included in the formulary. There is a limited number of antibiotics available to prescribers in public primary care, none of which are on the Reserve list. This was also reflected in our findings in terms of number of antibiotic molecules that constituted DU90% when compared to those in the private sector. A substantial proportion of antibiotics from the Watch category was reported from the private sector which may warrant further monitoring in terms of utilisation rates and the appropriateness. The increase in consumption of Watch antibiotics which was observed in most countries in the recent years might reflect the market share of antibiotics and rates of resistant infections within the country [6, 25]. Furthermore, implementation of the National Antibiotic Guidelines is stricter in the public sector while the practitioners in the private sector are at liberty to adopt whichever guidelines they see fit, be it from the local context or not. The national Antimicrobial Stewardship Programme (ASP) conducted by MOH is only currently enforced in government facilities. Private GPs are not subjected to the clinical and structural audits that are a part of the ASP.

The private sector accounted for a larger proportion of overall antibiotic use in primary care than the public sector in Malaysia. This finding echoes a previous cross-sectional study on antibiotic prescribing practice in primary care in Malaysia in 2014 [26]. The proportion of antibiotic utilisation in the private sector at the beginning of 2018 (six times higher than in public primary care clinics) was slightly lower than the results of a previous study in 2012 (seven times higher than in public primary care clinics) [27]. However, by the end of 2021, private primary care antibiotic utilisation was almost ten times higher than in public primary care, indicating an increasing trend in the proportion of antibiotic utilisation by the private sector. Higher antibiotic utilisation in the private sector is postulated to be due to a higher percentage of acute cases seen in private clinics [7, 28]. Furthermore, patients are more likely to demand antibiotics in the private sector due to greater out-of-pocket payments. Prescribers are then more inclined to give in to patient demands to supply antibiotics unnecessarily [29]. Sensitivity analyses looking at antibiotic utilisation in private clinics only indicated there was not much change in the results, only seeing a slight decrease in proportion of Access category antibiotics used and macrolides comprising a greater proportion of private sector utilisation of antibiotics. This indicates that the types of antibiotics used in retail pharmacies is fairly similar to that in private clinics.

Antimicrobial surveillance steps in Malaysia begun in 2019 with the implementation of specific terms of references and clinical pathways for antibiotic use in primary care [12]. Infection control measures for public primary care facilities also included hand hygiene protocols and audits [30]. Though most of the initiatives were largely conducted in the public health sector, efforts are being made to expand the program coverage for implementation at private facilities. The stewardship program includes the formation of a multidisciplinary team to conduct surveillance and feedback activities on antimicrobial consumption. Given the current structure of private primary care practices in Malaysia, collaborative partnerships between general practitioners and community pharmacists are necessary to promote quality use of not only antibiotics, but medicines in general [31–33]. Lower antibiotic consumption as well as increased guideline-adherent prescribing have also been documented when pharmacists were involved in the process [34–37].

### Limitations

Our study has limitations that need to be considered when interpreting the results. Data were only available from 2018, limiting analysis of earlier trends. We used aggregated procurement data for analysis which may not accurately reflect consumption of antibiotics. There may have been drugs procured but not utilised due to expiration or wastage. Data from private retail pharmacies might include antibiotics supplied for prescriptions from hospitals or veterinary purposes which we are not able to differentiate from this database. Finally, as the data are not available at individual patient-level, we are not able to study the appropriateness of antibiotics usage.

### Implications for research and/or practice

Our study contributes data on antibiotics utilisation rate at primary health care facilities in Malaysia and provides a better understanding on patterns of antibiotic use between public and private sector. Future work to look into prescription level data will shed more light on the appropriateness of antibiotic utilisation and potential overuse or misuse of antibiotics. This will enable us to better understand and evaluate the trends in antibiotic use, for instance, the increasing use of aminoglycosides, of which two of the antibiotics in the class are on the Watch list. Compared to neighbouring countries such as Singapore and Myanmar, Malaysia’s national research agenda on antimicrobials is still in its infancy [30]. This includes a budget allocation for research or collaborative work. A national research agenda will identify and highlight focus research areas for the country in order to inform antibiotic guidelines and policy. Targets for reducing total antibiotic utilisation rates and inappropriate prescribing has to be applied to both public and private sector against the setting of antimicrobial resistance goals.

## Conclusion

We described antibiotic usage across public and private primary care facilities in Malaysia over the period 2018–2021. We identified a substantial reduction in antibiotic utilisation rates, particularly during the COVID-19 pandemic period across both sectors. The patterns of antibiotic usage indicate greater variations in types of antibiotic molecules used in the private compared to the public sector. The WHO AWaRe classification provides a useful framework to measure antibiotic utilisation patterns and setting targets for antibiotic surveillance systems. Despite higher antibiotic utilisation rates noted in the private sector compared to their public counterparts, both sectors are in line with WHO’s target of at least 60% of antibiotic use consists of Access antibiotics. These findings highlight the need for more rigorous interventions, targeting both prescribers and the public. Improvement strategies should focus on reducing inappropriate and unnecessary prescribing.

### Electronic supplementary material

Below is the link to the electronic supplementary material.

Supplementary Material 1: List of Supplementary Figures and Tables

## Data Availability

Data and materials used in this study is not publicly available but can be provided upon reasonable request from the corresponding author (Audrey Huili Lim, audreylim@moh.gov.my).

## Abbreviations

DDD: Defined Daily Doses
DID: Defined Daily Doses per 1000 inhabitants
WHO: World Health Organisation
MOH: Ministry of Health Malaysia
AMS: Antimicrobial Stewardship
ATC: Anatomical Therapeutic Chemical
